# Independent inhibition of the polymerase and deubiquitinase activities of the Crimean-Congo Hemorrhagic Fever Virus full-length L-protein

**DOI:** 10.1371/journal.pntd.0008283

**Published:** 2020-06-04

**Authors:** Egor P. Tchesnokov, Ben A. Bailey-Elkin, Brian L. Mark, Matthias Götte

**Affiliations:** 1 Department of Medical Microbiology and Immunology, University of Alberta, Edmonton, Alberta, Canada; 2 Li Ka Shing Institute of Virology at University of Alberta, Edmonton, Alberta, Canada; 3 Department of Microbiology, University of Manitoba, Winnipeg, Canada; University of Texas Medical Branch, UNITED STATES

## Abstract

**Background:**

The Crimean-Congo hemorrhagic fever virus (CCHFV) is a segmented negative-sense RNA virus that can cause severe human disease. The World Health Organization (WHO) has listed CCHFVas a priority pathogen with an urgent need for enhanced research activities to develop effective countermeasures. Here we adopted a biochemical approach that targets the viral RNA-dependent RNA polymerase (RdRp). The CCHFV RdRp activity is part of a multifunctional L protein that is unusually large with a molecular weight of ~450 kDa. The CCHFV L-protein also contains an ovarian tumor (OTU) domain that exhibits deubiquitinating (DUB) activity, which was shown to interfere with innate immune responses and viral replication. We report on the expression, characterization and inhibition of the CCHFV full-length L-protein and studied both RNA synthesis and DUB activity.

**Methodology/Principle findings:**

Recombinant full-length CCHFV L protein was expressed in insect cells and purified to near homogeneity using affinity chromatography. RdRp activity was monitored with model primer/templates during elongation in the presence of divalent metal ions. We observed a 14-mer full length RNA product as well as the expected shorter products when omitting certain nucleotides from the reaction mixture. The D2517N mutation of the putative active site rendered the enzyme inactive. Inhibition of RNA synthesis was studies with the broad-spectrum antivirals ribavirin and favipiravir that mimic nucleotide substrates. The triphosphate form of these compounds act like ATP or GTP; however, incorporation of ATP or GTP is markedly favored over the inhibitors. We also studied the effects of bona fide nucleotide analogues 2’-deoxy-2’-fluoro-CTP (FdC) and 2’-deoxy-2’-amino-CTP and demonstrate increased inhibitory effects due to higher rates of incorporation. We further show that the CCHFV L full-length protein and the isolated OTU domain cleave Lys48- and Lys63-linked polyubiqutin chains. Moreover, the ubiquitin analogue CC.4 inhibits the CCHFV-associated DUB activity of the full-length L protein and the isolated DUB domain to a similar extent. Inhibition of DUB activity does not affect elongation of RNA synthesis, and inhibition of RNA synthesis does not affect DUB activity. Both domains are functionally independent under these conditions.

**Conclusions/Significance:**

The requirements for high biosafety measures hamper drug discovery and development efforts with infectious CCHFV. The availability of full-length CCHFV L-protein provides an important tool in this regard. High-throughput screening (HTS) campaigns are now feasible. The same enzyme preparations can be employed to identify novel polymerase and DUB inhibitors.

## Author summary

The tick-born Crimean-Congo hemorrhagic fever virus (CCHFV) causes severe human disease with high fatality rates. Outbreaks have been documented in a large geographic area from Africa to Asia. Unfortunately, vaccines that would prevent infection with the virus or antiviral drugs that can be administered for disease treatment are not available. Biosafety requirements further impede research in this area. The development of biochemical tools could potentially address this problem. Here we have expressed recombinant viral L-protein in insect cells. The L-protein is unusually large and exhibits RNA synthesis and deubiquitinating activities that are required for efficient viral growth. We have demonstrated that two distinct activities can be monitored in biochemical assays. Inhibition of these activities was shown with prototypic compounds. Hence, the purified L-protein provides an attractive target and tool for future drug discovery and development efforts.

## Introduction

The Crimean-Congo hemorrhagic fever virus (CCHFV) is a segmented negative-sense RNA virus that belongs to the Bunyavirales order, family *Nairoviridae* [1]. CCHFV is either transmitted directly from ticks to humans, indirectly via a wide range of infected animals or from person-to-person by contact with infectious body fluids. Infection can cause severe human disease and is endemic in Asia, the Middle East, Africa, and in parts of southern Europe [2]. CCHFV is viewed as an emerging threat at the Asian/European interface where Turkey reports ~700–1.300 cases per year [3]. Effective vaccines or treatments are not available. For these reasons, the World Health Organization (WHO) has listed CCHFV as a priority pathogen with an urgent need for enhanced Research & Development (R&D) activities [4].

Viral polymerases are validated drug targets. However, inhibition of the RNA-dependent RNA polymerase (RdRp) activity of negative-sense RNA viruses has been challenging. Previous drug discovery and development efforts focused on the influenza virus, which also contains a segmented genome [5–7]. The RdRp inhibitor favipiravir (T-705) is approved for the treatment of influenza in Japan [8]. This compound mimics the structure of a purine that is intracellularly activated to a nucleoside triphosphate (NTP) with a pseudo base. Biochemical studies with purified influenza RdRp demonstrated ambiguous base-pairing with both cytidine and uridine, which can lead to lethal mutagenesis or premature termination of RNA synthesis [6]. Similar mechanisms have earlier been proposed for the structurally related ribavirin [9]. Both drugs show a broad spectrum of activities against RNA viruses, including CCHFV [10–13]. Although some studies have indicated a clinical benefit, a small randomized trial did not reveal a significant difference between the groups of patients who received the drugs plus supportive treatment or supportive treatment only [14]. Evaluation of the drugs in mice models suggests that favipiravir is more potent when compared with ribavirin [11,12]. 2’-deoxy-2’-fluorocytidine (FdC) was also shown to inhibit CCHFV replication, and this compound seems to exceed the potency of both ribavirin and favipiravir [15]. FdC was originally identified as an inhibitor of hepatitis C virus (HCV) replication, but its development as an antiviral drug has been limited due to off-target activities [16].

**Fig 1:**
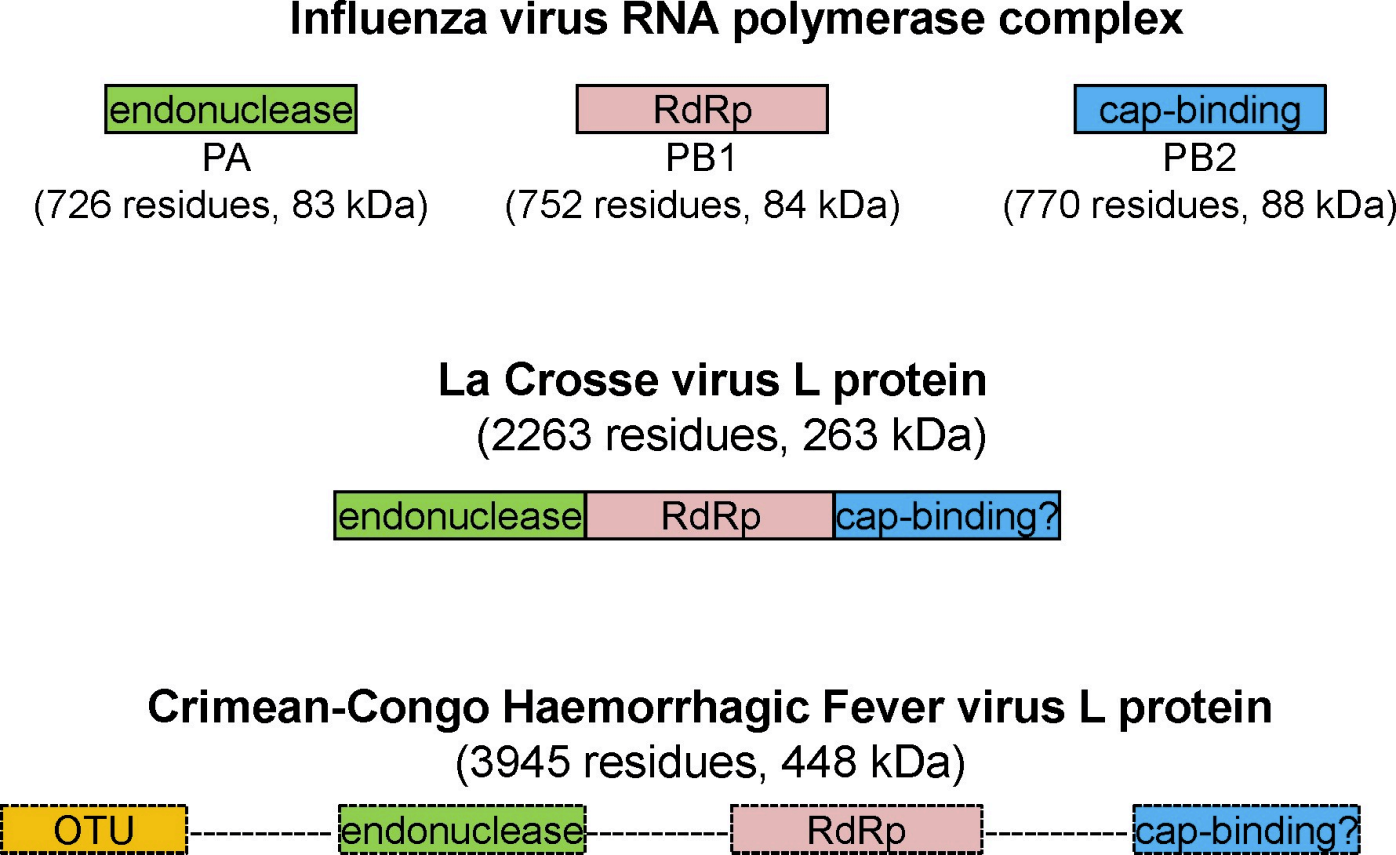
Functional domains in L-proteins and related complexes of segmented negative-sense RNA viruses. The RNA polymerase complex of influenza contains three subunits with the following functions: cap-snatching endonuclease (PA; 726 residues, 83 kDa), RdRp (PB1; 752 residues, 84 kDa) and cap-binding (PB2; 770 residues, 88 kDa). Functional equivalent domains are evident in the monomeric L-protein (2263 residues, 263 kDa) of the La Crosse Virus although the cap binding function remains elusive. The L-protein (3945 residues, 448 kDa) of the Crimean-Congo Haemorrhagic Fever Virus contains an additional OTU domain at its N-terminus. The structural information for the CCHFV L protein is limited and is indicated by the dotted boxes and lines. The sizes of the functional domains are not shown in scale.

Biochemical tools that facilitate screening for CCHFV RdRp inhibitors have yet to be developed. The CCHFV RdRp activity is part of a multifunctional L protein that is currently not available for biochemical or structural studies [17,18]. The study of enzymes from other segmented negative-sense RNA viruses provided important insight into structure and function (Fig 1). The polymerase of influenza (*Orthomyxoviridae*) is a heterotrimeric complex composed of an RdRp subunit, a cap-snatching endonuclease, and a cap-binding domain (Fig 1, top) [19,20]. The structure of the La Crosse Virus (LACV) RdRp shows a similar overall arrangement in a single chain (Fig 1, middle) [21]. LACV belongs to the *Peribunyaviridae* family and is therefore more closely related to the CCHFV. The full-length CCHFV L-protein is unusually large with ~4000 amino acids and a molecular weight of ~450 kDa, which is commonly not conducive to heterologous expression. RdRp and Cap-snatching endonuclease motifs have been identified [22], although recent attempts to express the L protein or the isolated nuclease domain have failed to yield active enzyme [23]. Recently, a functional cap-binding domain has been identified in the C-terminus of the Rift Valley fever virus (RVFV) L protein [24]; however it is still unknown whether it is also present in the CCHFV L protein. The CCHFV L-protein also contains an ovarian tumor (OTU) domain that exhibits deubiquitinating (DUB) activity (Fig 1, bottom). Structural information is limited to the isolated CCHFV OTU domain [25]. The CCHFV OTU domain is a cysteine protease capable of removing ubiquitin (Ub) from proteins, which interferes with innate immune responses [10,26]. The ubiquitin variant CC.4 binds to the OTU domain with high affinity and inhibits CCHFV DUB activity [27]. CC.4 was also shown to affect infectivity and replication [28]. Hence, the availability of recombinant CCHFV full-length L protein would provide an important tool for drug discovery and development efforts.

Here, we utilized the baculovirus expression system and generated active full-length CCHFV L protein. RdRp activity is seen with model RNA substrates in the presence of catalytic, divalent metal ions. We confirm that the triphosphate (TP) forms of ribavirin, favipiravir, and 2’-deoxy-2’-fluoro-CTP are recognized as substrates and inhibit RNA synthesis. Moreover, we identified 2’-deoxy-2’-amino-CTP as a novel inhibitor of CCHFV RdRp. We further show that CC.4 inhibits the CCHFV-associated DUB activity of the full-length L protein and the isolated DUB domain to a similar extent. Inhibition of RdRp activity does not affect DUB activity and inhibition of DUB activity does not affect RdRp activity, which provides strong evidence to show that both domains are functionally independent.

## Methods

### Chemicals

All RNA primers and templates used in this study were purchased from Dharmacon (Lafayette, CO, USA). Ribavirin-TP was purchased from Jena Bioscience (Jena, Germany). Favipiravir-TP was purchased from Santa Cruz Biotechnology (Santa Cruz, CA, USA). 2’-deoxy-2’-fluoro-CTP (FdC-TP) and 2’-deoxy-2’amino-CTP (2’d-2’amino-CTP) were purchased from TriLink (San Diego, CA, USA). NTPs were purchased from GE Healthcare (Cranbury, NJ, USA). [α-^32^P]-GTP and–CTP were purchased from PerkinElmer (Boston, MA, USA). C-terminus derived ubiquitin conjugated with 7-amido-4-methylcoumarin (ubiquitin-AMC) was purchased from Boston Biochem (Cambridge, MA, USA).

### Protein expression and purification

The pFastBac-1 (Invitrogen, Burlington, ON, Canada) plasmid with the codon-optimized synthetic DNA sequences (GenScript, Piscataway, NJ, USA) coding for CCHFV L protein (AIE16126) with N-terminal Strep- and 8xhistidine (His)-tags was used as a starting material for protein expression in insect cells (Sf9, Invitrogen, Burlington, ON, Canada). We employed the MultiBac (Geneva Biotech, Indianapolis, IN, USA) system for protein expression in insect cells (Sf9, Invitrogen, Burlington, ON, Canada) according to published protocols [29,30]. CCHFV L protein was initially purified using the strep- or his-tag-affinity chromatography according to the manufacturer’s specifications (IBA, (Goettingen, Germany), and Thermo Scientific, Rockford, IL, USA, respectively). His-tag purification yielded generally higher amounts of protein. The identity of the purified CCHFV L protein was confirmed by mass spectrometry (MS) analysis (Dr. Jack Moore, Alberta Proteomics and Mass Spectrometry, Edmonton, AB, Canada). The mass spectrometry peptide fragments included residues starting from N6 to R3935 out of 3945 residues of the full length CCHFV L protein, which corresponds to 99.6% coverage.

### Purification of the isolated CCHFV OTU domain

Residues 1–217 of the CCHFV L protein were inserted into vector pET-49b(+) and expressed as a GST-tagged fusion. The CCHFV OTU domain was purified form *Escherichia coli*, and the GST tag removed using methods describe previously [25]. Expression and purification of CC.4 was performed as previously described [27].

### RNA synthesis activity

Data acquisition and quantification were done as previously reported by us [31,32]. The amount of CCHFV L used in the RNA synthesis assay was optimized such that incorporation of [α-^32^P]-NTP (PerkinElmer, Boston, MA, USA) would reach its maximum after 30 min. RNA synthesis assay consisted of mixing (final concentrations) Tris-HCl (pH 8, 25 mM), RNA primer (200 μM), RNA template (1 μM), [α-^32^P]-NTP (0.1 μM), various concentrations and combinations of 100 μM (or as indicated) NTP and NTP analogues, and CCHFV L (50–100 nM) on ice. Reaction mixtures (10 μL) were incubated for 10 min at 30°C followed by the addition of 5 μL of MgCl_2_ (5 mM). Reactions were stopped after 30 min by the addition of 15 μL of formamide/EDTA (50 mM) mixture and incubated at 95°C for 10 min. 3 μL reaction samples were subjected to denaturing 8 M urea 20% polyacrylamide gel electrophoresis to resolve products of RNA synthesis followed by signal quantification (ImageQuant 5.2, GE Healthcare Bio-Sciences, Uppsala, Sweden) through phosphorimaging (Typhoon TRIO variable mode imager, GE Healthcare Bio-Sciences, Uppsala, Sweden). Incorporated nucleotide product fraction was plotted versus nucleotide substrate concentrations and fitted to the Michaelis-Menten equation using GraphPad Prism 7.0 (GraphPad Software, Inc., San Diego, CA, USA). Protein preparations were stored at -20˚C in 40% glycerol solution containing 67 mM Tris-HCl (pH 8), 100 mM NaCl, 5 mM TCEP, 0.05% Tween-20 and remained active for the duration of the study.

### Deubiquitinase (DUB) activity of CCHFV L protein and the isolated OUT domain

#### Qualitative analysis of polyUb chain hydrolysis using SDS-PAGE

600 ng of either K48- or K63-linked polyubiquitin chains (Ub_3_-Ub_7_; Boston Biochem) were incubated with either purified CCHFV L protein, or CCHFV OTU_(1–217)_ at a final concentration of 20 nM in a final volume of 20 μL. The CCHFV OTU-specific inhibitor CC.4 was incubated with CCHFV L protein or OTU_(1–217)_ at a final concentration of 100 nM. Reaction were performed in in 50 mM Tris pH 8.5, 100 NaCl, 5 mM DTT at 37°C. At pre-determined time points, 6 μL of each reaction were removed stopped with the addition of 2X SDS-PAGE loading buffer. Reactions were resolved by electrophoresis on a 10% Tris-Tricine gel and visualized using a Pierce silver stain kit.

#### Quantitative analysis of DUB activity using fluorescene-based assay

A C-terminus-derived ubiquitin peptide conjugated with 4-amino-4-methylcoumarin (AMC) was used as a fluorogenic substrate for 5 nM CCHFV L protein or isolated OTU domain in a reaction buffer containing Tris-HCl (pH 8, 25 mM) and TCEP (2 mM). Reactions were started by the addition of ubiquitin-AMC (500 nM) in a final reaction volume of 25 μL. Reactions were conducted in a black 384-well plate (Greiner-784900, Greiner Bio-One, Kremsmünster, Austria).

Once AMC is cleaved from the conjugated substrate it can emit fluorescence at 445 nm upon excitation at 355 nm acquired on a SpectraMax M5 (Molecular devices, San Jose, CA, USA). Slopes of linear portions of fluorescent signal formation were used to determine the velocity of substrate cleavage in the presence and absence of ubiquitin variant CC.4 (CC.4) which has been shown previously to inhibit DUB activity of CCHFV isolated OTU-domain [27]. DUB activity velocities were plotted versus CC.4 concentrations and fitted to a log(inhibitor)-versus-normalized-response-(variable slope) equation using GraphPad Prism 7.0 (GraphPad Software, Inc., San Diego, CA, USA) to determine the IC_50_ values for the inhibition of DUB activity by CC.4.

## Results

### Purification and RNA synthesis activity of CCHFV L full length protein

**Fig 2:**
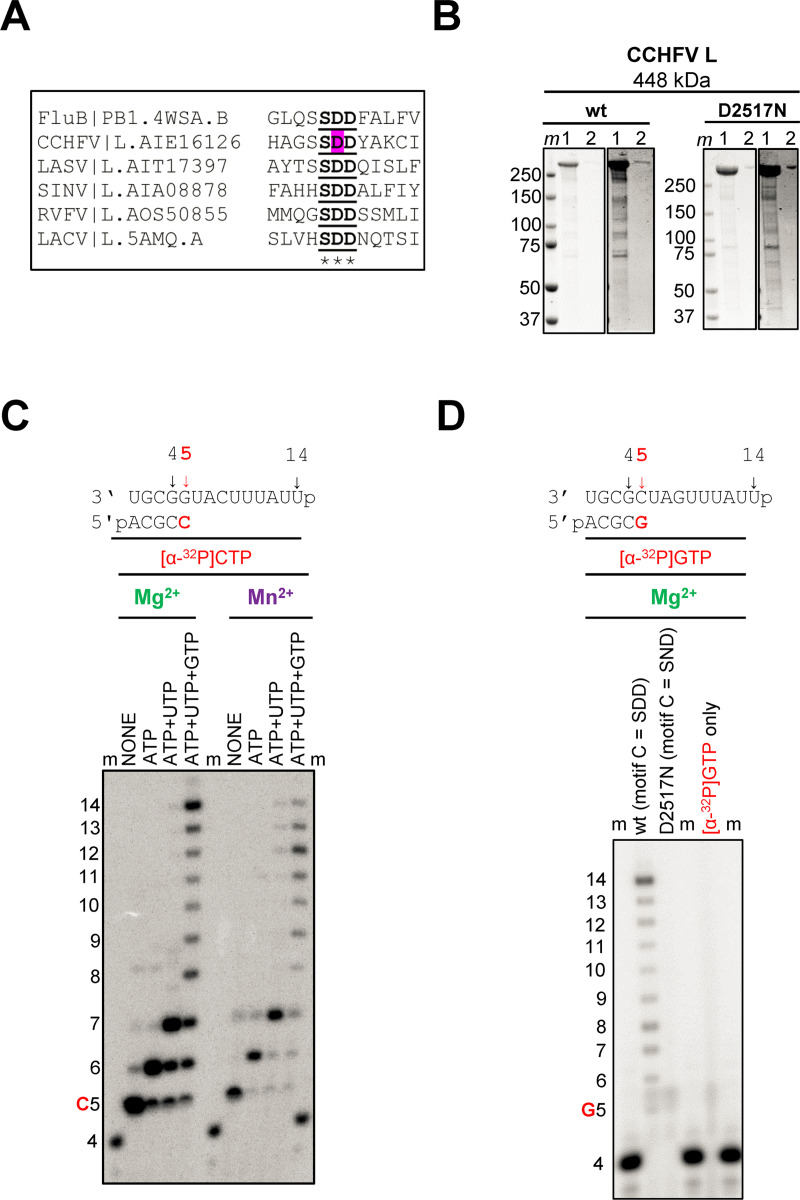
Purification and characterization of the RdRp activity of recombinant CCHFV L wild type and D2517N mutant proteins. (**A**) A partial sequence alignment of RdRp of segmented viruses shows the conserved SDD sequence within motif C. Residue D2517 within CCHFV L protein is highlighted in purple. (**B**) SDS PAGE migration pattern of the purified enzyme preparation stained with Coomassie Brilliant Blue G-250 dye. The band migrating above the 250 kDa molecular weight marker (lane 1) contains CCHFV L wild type or D2517N mutant full-length protein (as indicated) confirmed by mass spectrometry (see S1 and S2 Figs for details). Lane 2 contains 20x less protein than lane 1. The protein band is still visible, suggesting that there is ~20 times more of the CCHFV L protein than impurities. This corresponds to ~95% pure protein preparation. (**C**) RNA primer/template used in the RNA synthesis assays is shown above the gel. Template and primer were both mono-phosphorylated (p) at their 5’-ends. C indicates incorporation of the radiolabeled nucleotide opposite template position 5. RNA synthesis was monitored with purified wild type CCHFV L protein in the presence of [α-^32^P]CTP, RNA primer/templates and NTP combinations designed to generate either intermediate or full template length products. Note that 10x less of wild type CCHFV L protein was used in reactions started with MnCl_2_. Lane m illustrates the migration pattern of the radiolabeled 4 nucleotide-long primer. (**D**) RNA primer/template used in the RNA synthesis assays is shown above the gel. Template and primer were both phosphorylated (p) at their 5’-ends. G indicates incorporation of the radiolabeled nucleotide opposite template position 5. RNA synthesis was monitored with purified CCHFV L wt and D2517N mutant proteins in the presence of [α-^32^P]GTP, RNA primer/template and NTP combinations designed to generate full template length products. Lane m illustrates the migration pattern of the radiolabeled 4 nucleotide-long primer. Lane “[α-^32^P]GTP-only’ illustrates the background signal associated with the [α-^32^P]GTP preparation in the absence of enzyme.

The baculovirus expression system has been successfully used to generate recombinant L-protein from segmented negative-sense RNA viruses, including influenza B and La Crosse [19,21]. Here we employed this approach to express recombinant CCHFV L protein with an N-terminal His-tag (Fig 2). We have also generated an active site mutant that contains a D to N change at position 2517. The aspartic residue is located in motif C and is responsible for coordinating the catalytic metal ions [31,33,34]. Motif C is conserved among RdRp enzymes of segmented RNA viruses (Fig 2A). SDS-PAGE analysis of the protein preparation revealed a band above the 250 kDa molecular weight marker (Fig 2B), which corresponds to the full-length CCHFV L protein. Both proteins were purified to near homogeneity and unambiguously identified through mass spectrometry (Methods, S1 and S2 Figs).

Initiation of RNA synthesis involves conserved sequences at the 5’- and 3’-untranslated regions of the RNA genome as demonstrated for influenza and other segmented negative sense RNA viruses [19,20,35]. Transitioning to elongation follows after a few nucleotide additions. The elongation mode is largely independent of the sequence and facilitates the study of RNA synthesis inhibition with nucleotide analogues. Hence, we devised short model primer/templates that adequately mimic the elongation state. Originally used for the study of respiratory syncytial virus (RSV) RdRp [36], these type of sequences were recently utilized to quantify nucleotide incorporation events with various RdRp enzymes from Ebola virus (EBOV), hepatitis C virus (HCV), Zika virus (ZIKV), influenza B virus (FluB), as well as Middle East respiratory syndrome coronavirus (MERS-CoV) [31,34]. In this study we employed the same biochemical approach for comparative purpose. The 14-mer template (Fig 2C) is designed such that [α-^32^P]CTP is utilized as the first nucleotide substrate and incubation with specific NTP cocktails would be expected to yield defined reaction products. The data show that Mg^2+^ as well as Mn^2+^ can serve as catalytic metal ions and promote extensions of a 4-mer primer. Limited RNA synthesis in the presence of [α-^32^P]CTP alone revealed the expected 5-mer product. The presence of [α-^32^P]CTP and ATP yields a 6-mer product, a mixture of [α-^32^P]CTP, ATP and UTP allows formation of a 7-mer and the addition of all four NTPs yields a 14-mer full-length RNA product. Template-independent nucleotide additions are not observed. Mg^2+^ was used throughout this study as this is the preferred ion with respect to the yield of the full-length product. The D2517N active site mutant was inactive when tested for RNA synthesis (Fig 2D).

### Inhibition of CCHFV RdRp with Favipiravir and Ribavirin

**Fig 3:**
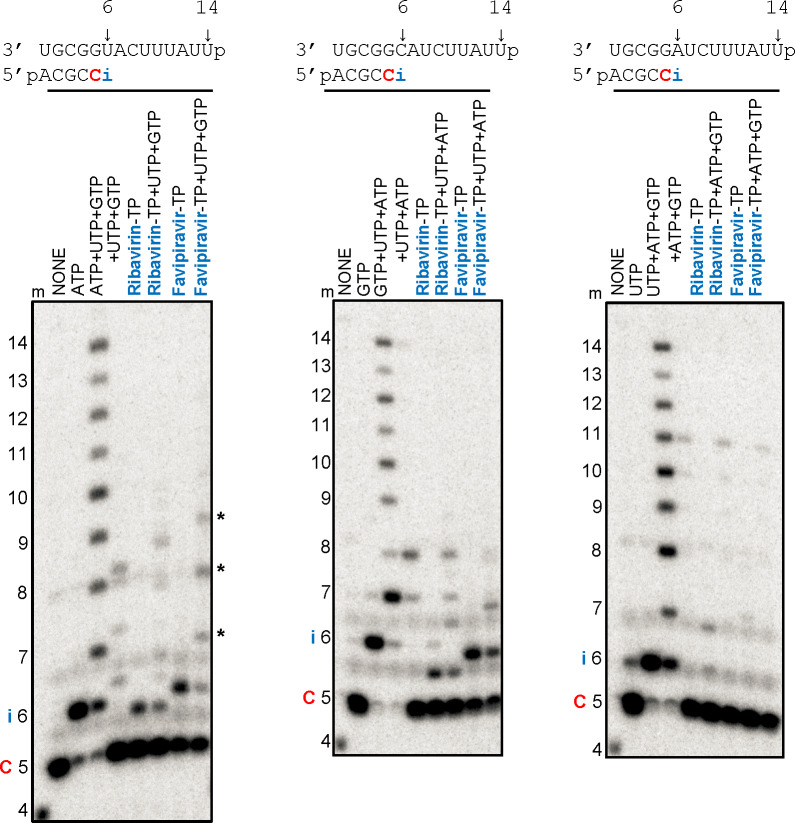
Patterns of inhibition of RNA synthesis with Ribavirin- and Favipiravir-TP. (**A**) RNA primer/templates used in the RNA synthesis assays to test incorporation of Ribavirin-MP and Favipiravir-MP as A-, G-, and UMP-analogues are shown above the respective gels. C indicates incorporation of the radiolabeled nucleotide opposite template position 5. Position i allows incorporation of A-, G-, and UMP or nucleotide analogue inhibitors. Ribavirin-MP and Favipiravir-MP incorporation as A-, G-, and UMP-analogues was monitored with purified CCHFV L protein in the presence of [α-^32^P]GTP, RNA primer/template, 5 mM MgCl_2_ and various combinations of 100 μM NTP and 100 μM NTP substrate analogues. The presence of three natural NTPs allows full-length product formation up to position 14. The presence of two natural NTPs provides a control for mis-incorporations. Lane m illustrates the migration pattern of the radiolabeled 4 nucleotide-long primer. Asterisks indicate limited ongoing primer extensions.

Previous biochemical studies have shown that both favipiravir-TP and ribavirin-TP are substrates for several RdRp enzymes and compete with ATP or GTP for incorporation into the growing RNA chain [5,6,9,37]. Here we studied possible effects of these inhibitors on RNA synthesis by CCHFV RdRp (Fig 3). We employed a 4-mer primer and three different RNA templates to assess efficiency of incorporation opposite template U, C, and A at position 6 (Fig 3, top). Limited RNA synthesis in the presence of [α-^32^P]CTP yields the labeled 5-mer product for all three templates. The presence of [α-^32^P]CTP and ATP yields the expected 6-mer product RNA and the presence of all four NTPs allows full-length product formation (Fig 3, left). The omission of ATP resulted in minor abortive products that likely reflect limited mis-incorporations and extensions. The same reactions were performed with ribavirin-TP and favipiravir-TP, respectively. The presence of [α-^32^P]CTP and ribavirin-TP, instead of ATP, also yields a 6-mer product. The additional presence of UTP and GTP shows limited ongoing primer extensions. Full-length product formation is not observed under these conditions, which indicates inhibition of RNA synthesis. We obtained similar results with favipiravir-TP. Both ribavirin-TP or favipiravir-TP are also used as substrates opposite template C (Fig 3, middle). In contrast, the inhibitors are not incorporated opposite template A (Fig 3, right). Together the data show that CCHFV RdRp incorporates ribavirin and favipiravir opposite template U and C as described for other RdRp enzymes. Following incorporation of these compounds, RNA synthesis is severely compromised. Partial chain-termination is a likely mechanism of inhibition.

**Fig 4:**
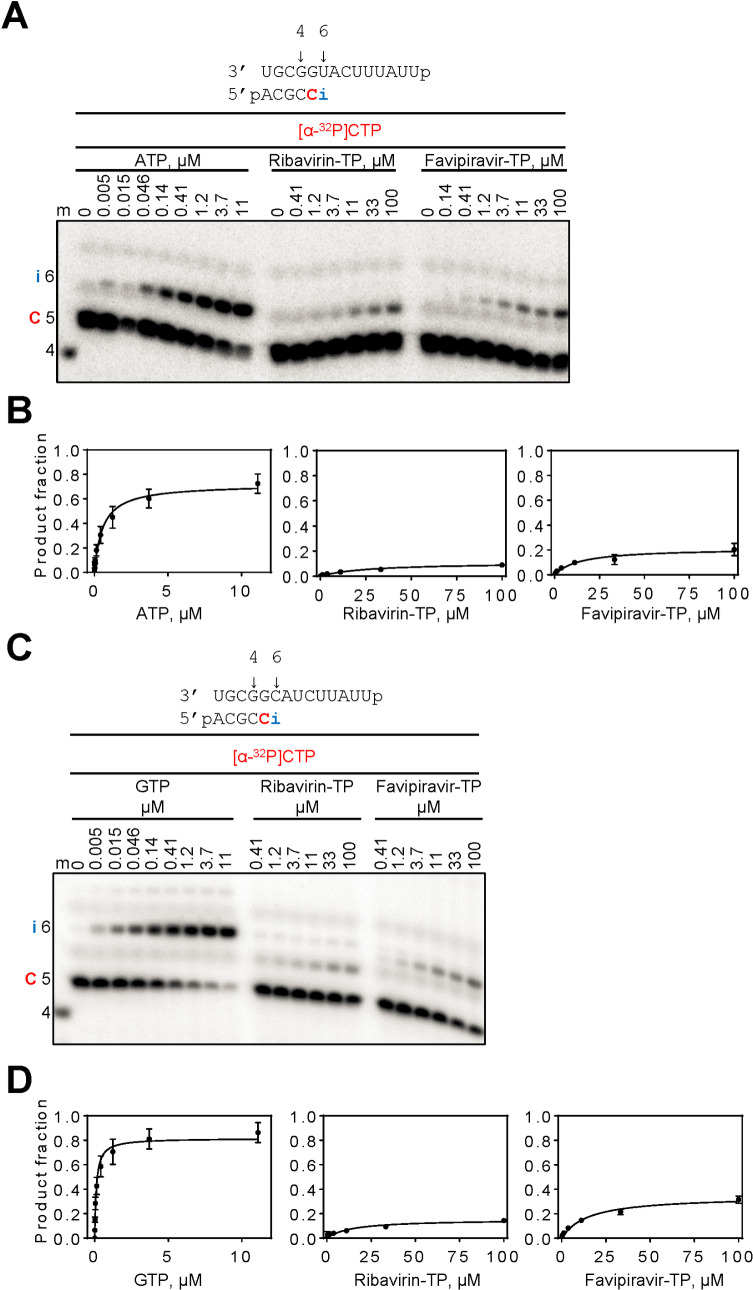
Selective incorporation of ribavirin- and favipiravir-MP opposite template U and C. (**A, C**) RNA primer/template used in the RNA synthesis assays to test incorporation of ribavirin-MP and favipiravir-MP as AMP or GMP-analogues are shown above the respective gels. C indicates incorporation of the radiolabeled nucleotide opposite template position 5. Position i allows incorporation of AMP or GMP or nucleotide analogue inhibitors. NTP incorporation was monitored with purified CCHFV L protein in the presence of [α-^32^P]CTP, RNA primer/template, 5 mM MgCl_2_ and increasing concentrations of NTP and NTP substrate analogues. Lane m illustrates the migration pattern of the radiolabeled 4 nucleotide-long primer. (**B**) Graphic representation of the data for incorporation of AMP and ribavirin- or favipiravir-MP opposite template U. (**C**) Graphic representation of the data for incorporation of GMP and ribavirin- or favipiravir-MP opposite template C. Error bars represent standard deviation of data from three independent experiments.

Incorporation and in turn inhibition by ribavirin-TP or favipiravir-TP requires that the nucleotide analogues are able to compete with ATP or GTP. Quantitative data can be obtained by measuring enzyme kinetic parameters and compare the efficiency of incorporation of the inhibitor with their natural counterparts. For this purpose, the natural nucleotide or the inhibitor was added at increasing concentrations at a fixed time point following the addition of [α-^32^P]CTP (Fig 4A and 4C). A graphic representation of the data is shown in Fig 4B and 4D. It is evident that product formation is much more efficient with the natural substrates ATP and GTP. Steady-state kinetic parameters *K*_m_ and *V*_max_ are provided in Table 1. Favipiravir-TP and ribavirin-TP show reduced *V*_max_ and increased *K*_m_ values when compared with ATP or GTP. Both factors lead to reductions in the efficiency of incorporation *V*_max_ / *K*_m_. The selectivity of nucleotide incorporation is defined as *V*_max_ / *K*_m_ (NTP)/ *V*_max_ / *K*_m_ (inhibitor) and helps to translate these values into quantitative terms. The selectivity values for ribavirin are relatively high when measured against ATP (304) and GTP (696), respectively. The values for favipiravir are slightly more favorable against both ATP (86) and GTP (329). Together, the data suggest that the incorporation of ATP or GTP is markedly favored over the analogues, which could limit their antiviral effects.

**Table 1:** CCHFV L protein selectivity values for Ribavirin-TP and Favipiravir-TP.

		Inhibitor			Inhibitor	
	ATP	Ribavirin -TP	Favipiravir -TP	GTP	Ribavirin -TP	Favipiravir -TP
***V***_**max**_^a^ (product fraction)	**0.72**^d^	**0.11**	**0.21**	**0.82**	**0.15**	**0.34**
*±*	0.032^e^	0.009	0.021	0.026	0.016	0.018
% error ^f^	4	8	10	3	11	5
***K***_**m**_ ^b^ (μM)	**0.56**	**26**	**14**	**0.11**	**14**	**15**
*±*	0.099	5	4.4	0.018	5	2.5
% error	18	21	31	16	34	17
***V***_**max**_**/ *K***_**m**_	1.3	0.004	0.02	7.5	0.011	0.02
**Selectivity**^c^ (fold)	1^f^	**304**	**86**	1	**696**	**329**

^*a*^ *V*_max_ is a Michaelis–Menten parameter reflecting the maximal velocity of nucleotide incorporation.

^*b*^ *K*_m_ is a Michaelis–Menten parameter reflecting the concentration of the nucleotide substrate at which the velocity of nucleotide incorporation is half of *V*_max._

^*c*^ Selectivity of a viral RNA polymerase for a nucleotide substrate analogue is calculated as the ratio of the *V*_max_/*K*_m_ values for NTP and NTP analogue, respectively.

^*d*^ All reported values have been calculated on the basis of a 9-data point experiment repeated three times (n = 3) for natural NTP substrate and the substrate analogue

^*e*^ Standard error associated with the fit.

^*f*^Reference.

### Inhibition of CCHFV RdRp with bona fide nucleotide analogues

**Fig 5:**
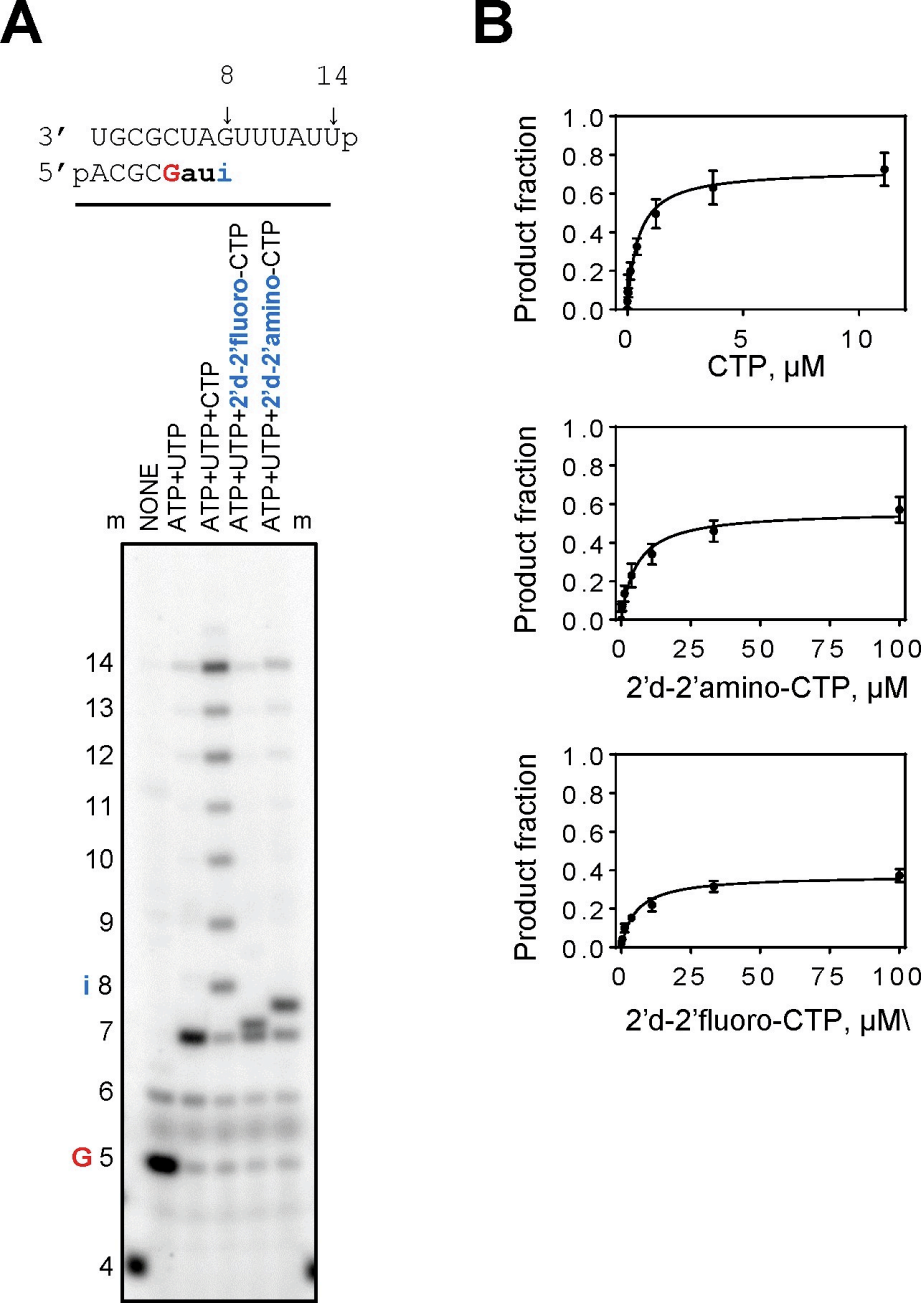
Patterns of inhibition of RNA synthesis with 2’deoxy-2’-fluoro-CTP and 2’deoxy-2’-amino-CTP. (**A**) RNA primer/template used in the RNA synthesis assays to test incorporation of with 2’deoxy-2’-fluoro- CTP and 2’deoxy-2’-amino- CTP as a CMP-analogue across templating G at position 8 is shown above the gel. G indicates incorporation of the radiolabeled nucleotide opposite template position 5. Position i allows incorporation of CMP or nucleotide analogue inhibitors. 2’deoxy-2’-fluoro- CTP and 2’deoxy-2’-amino- CTP incorporation was monitored with purified CCHFV L protein in the presence of [α-^32^P]GTP, RNA primer/template, 5 mM MgCl_2_ and various combinations of 100 μM NTP and 100 μM NTP substrate analogues. The presence of three natural NTPs allows full-length product formation up to position 14. The presence of two natural NTPs provides a control for mis-incorporations across templating G at position 8. Lane m illustrates the migration pattern of the radiolabeled 4 nucleotide-long primer. (**B**) Graphic representation of the data for selective incorporation of 2’deoxy-2’-fluoro-CMP and 2’deoxy-2’-amino-CMP. Error bars represent standard deviation of data from three independent experiments.

We next studied the effects of bona fide nucleotide analogues with an intact base moiety. As a starting point, we selected two compounds with bioisosteric replacements for the 2’- hydroxyl group that is commonly recognized by RdRp enzymes: 2’-deoxy-2’-fluoro-CTP (FdC) and 2’-deoxy-2’-amino-CTP. The 2’-fluoro and the 2’-amino groups may not prevent incorporation, but could potentially inhibit RNA synthesis. We devised an RNA template that allowed us to monitor incorporation of these compounds at position 8 (Fig 5A, top). Limited elongation of the 4-mer primer in the presence of [α-^32^P]GTP, ATP and UTP yields a 7-mer product and the addition of CTP allows formation of the expected 14-mer full-length RNA product (Fig 5A). The same reactions were also performed with FdC-TP and 2’-amino-CTP, respectively. Both compounds are incorporated to yield 8-mer products. Further primer extensions and full-length product formation is substantially reduced.

Kinetic parameters and selectivity values are provided in Table 2. Reductions in *V*_max_ are not as pronounced as seen with ribavirin-TP or favipiravir-TP. The main contribution to overall reductions in the efficiency of incorporation is here driven primarily by moderate increases in *K*_m_ values. As a consequence, selectivity values for 2’-deoxy-2’-fluoro-CTP (23) and 2’-deoxy-2’-amino-CTP (15) are relatively small when compared with ribavirin-TP or favipiravir-TP. Thus, the bona fide nucleotide analogues are better substrates for CCHFV RdRp.

**Fig 6:**
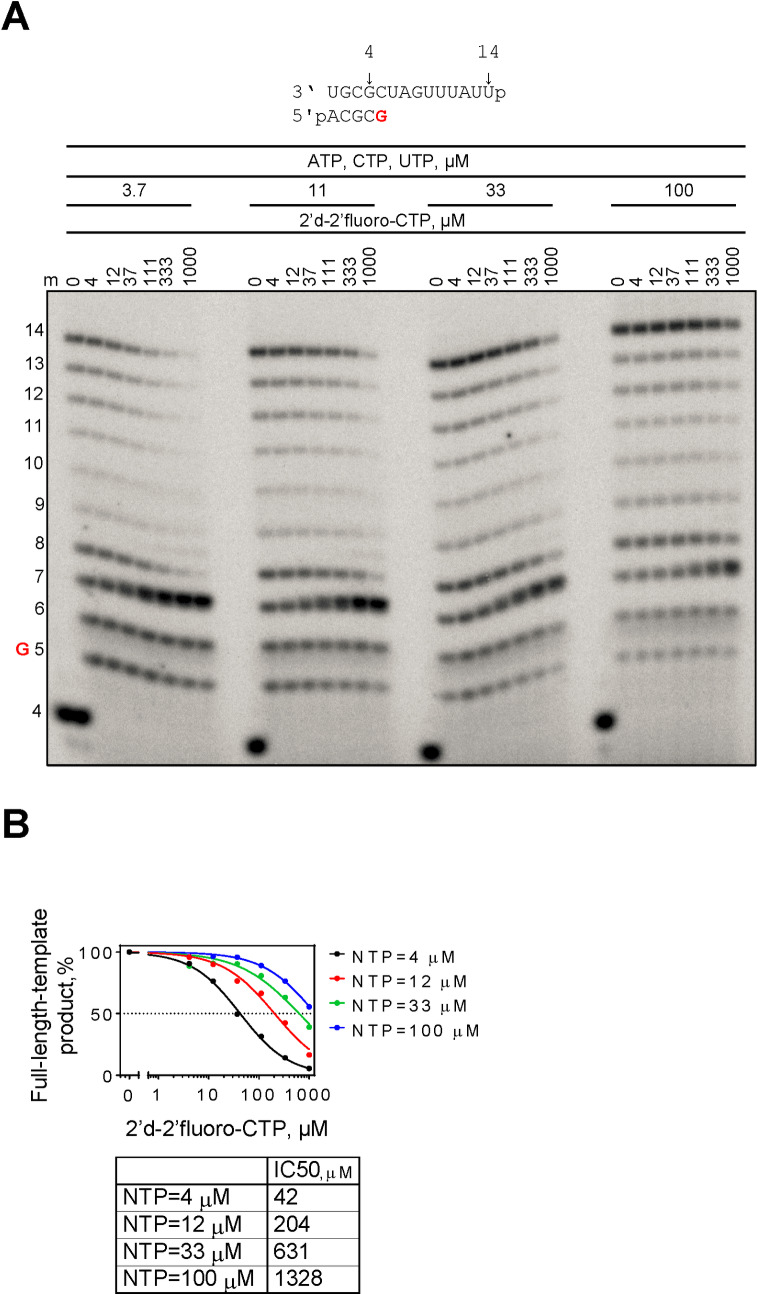
Competition between CTP and 2’deoxy-2’fluoro-CTP. (**A**) RNA primer/template used in the RNA synthesis assays to test competition of CTP and 2’deoxy-2’fluoro-CTP is shown above the gel. G indicates incorporation of the radiolabeled nucleotide opposite template position 5. Template G allows incorporation of CTP or 2’deoxy-2’fluoro-CTP and their competition for incorporation when both nucleotides are present in the reaction mixture. RNA synthesis was monitored with purified CCHFV L protein in the presence of [α-^32^P]GTP, RNA primer/template, 5 mM MgCl_2_ and 3.7, 11, 33 and 100 μM ATP, CTP, UTP and increasing concentrations of 2’deoxy-2’fluoro-CTP. The presence of three natural NTPs in the absence of 2’deoxy-2’fluoro-CTP allows full-length product formation up to position 14. Lane m illustrates the migration pattern of the radiolabeled 4 nucleotide-long primer. (**B**) Full-length-template product was quantified as a fraction of total signal in the lane, normalized to the full-template-length product fraction in the absence of 2’deoxy-2’fluoro-CTP and plotted versus log concentrations of the 2’deoxy-2’fluoro-CTP. Data were fitted to a dose response function in GraphPad (Prism 6.0) to determine the concentration of 2’deoxy-2’fluoro-CTP at which the amount of full-length-template product decreased by 50% (IC_50_).

We also tested incorporation and inhibition with 2’-deoxy-2’-fluoro-CTP in the presence of its natural competitor CTP (Fig 6A). The extent of 2’-deoxy-2’-fluoro-CMP incorporation and subsequent chain-termination of RNA synthesis depends on the competing CTP concentration as illustrated by increasing IC_50_ values for 2’-deoxy-2’-fluoro-CMP (Fig 6B).

**Table 2:** CCHFV L protein selectivity values for 2'amino-CTP and 2'fluoro-CTP.

	CTP	2'amino-CTP	2'fluoro-CTP
***V***_**max**_^a^ (product fraction)	**0.72** ^d^	**0.57**	**0.37**
±	0.032 ^e^	0.029	0.015
% error ^f^	4	5	4
***K***_**m**_ ^b^ (μM)	**0.46**	**6**	**5.4**
±	0.084	1.10	0.85
% error	18	20	16
***V***_**max**_**/ *K***_**m**_	1.6	0.10	0.07
**Selectivity**^c^ (fold)	1 ^g^	**15**	**23**

^*a*^ *V*_max_ is a Michaelis–Menten parameter reflecting the maximal velocity of nucleotide incorporation.

^*b*^ *K*_m_ is a Michaelis–Menten parameter reflecting the concentration of the nucleotide substrate at which the velocity of nucleotide incorporation is half of *V*_max._

^*c*^ Selectivity of a viral RNA polymerase for a nucleotide substrate analogue is calculated as the ratio of the *V*_max_/*K*_m_ values for NTP and NTP analogue, respectively.

^*d*^ All reported values have been calculated on the basis of a 9-data point experiment repeated three times (n = 3) for natural NTP substrate and the substrate analogue

^*e*^ Standard error associated with the fit.

^*f*^ Percent error.

^*g*^ Reference.

### Deubiquitinating activity and inhibition

**Fig 7:**
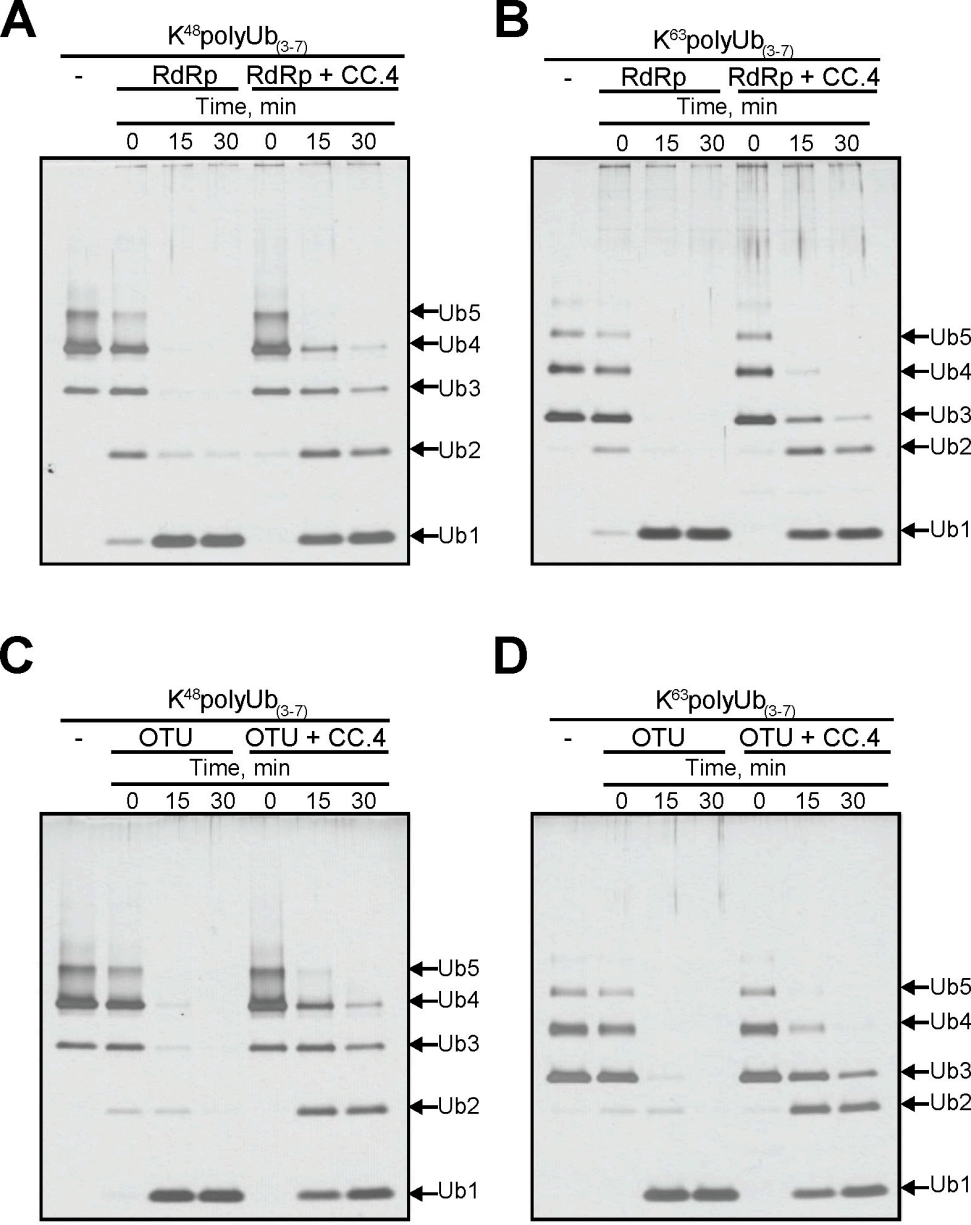
Qualitative analysis of polyUb chain hydrolysis by CCHFV L protein and the isolated CCHFV OTU domain. Purified CCHFV L protein (RdRp) was incubated with either (**A**) K^48^polyUb or (**B**) K^63^polyUb chains in the presence or absence of OTU-specific inhibitor CC.4. Ub chain hydrolysis was also assessed for the purified OTU domain using (**C**) K^48^polyUb chains or (**D**) K^63^polyUb chains in the presence or absence of CC.4. Reactions were incubated at 37°C and samples taken at 0, 15 and 30 minutes as indicated. Ub chain lengths following digestion are indicated with arrows. Samples were resolved on a 10% tris-tricine gel and visualized by silver stain.

The CCHFV-associated DUB activity was previously studied exclusively with the isolated OTU domain. Crystal structures of the viral OTU domain bound to ubiquitin provided important information about the active site and substrate binding [25]. Requirements for DUB activity and its inhibition in this context may now be compared with the full-length L protein. For an initial qualitative comparison of the two enzymes, we assessed deubiquitination with Lys48- and Lys63-linked polyubiqutin chains (Fig 7). The starting materials contains mixtures of polymers with 3–7 ubiquitin units. Time course experiments show that the full-length protein is able to process the polymeric chains to monomeric units. Similar results were obtained with Lys48-linked (Fig 7A, left) and Lys63-linked substrates (Fig 7B, left). Inhibition of these reactions was assessed with the CCHFV OTU-specific inhibitor CC.4 [27]. These conditions yield longer products predominantly with 1–4 ubiquitin units (Fig 7A and 7B, right). Most importantly, we obtained almost identical results with the isolated OTU domain (Fig 7C and 7D).

**Fig 8:**
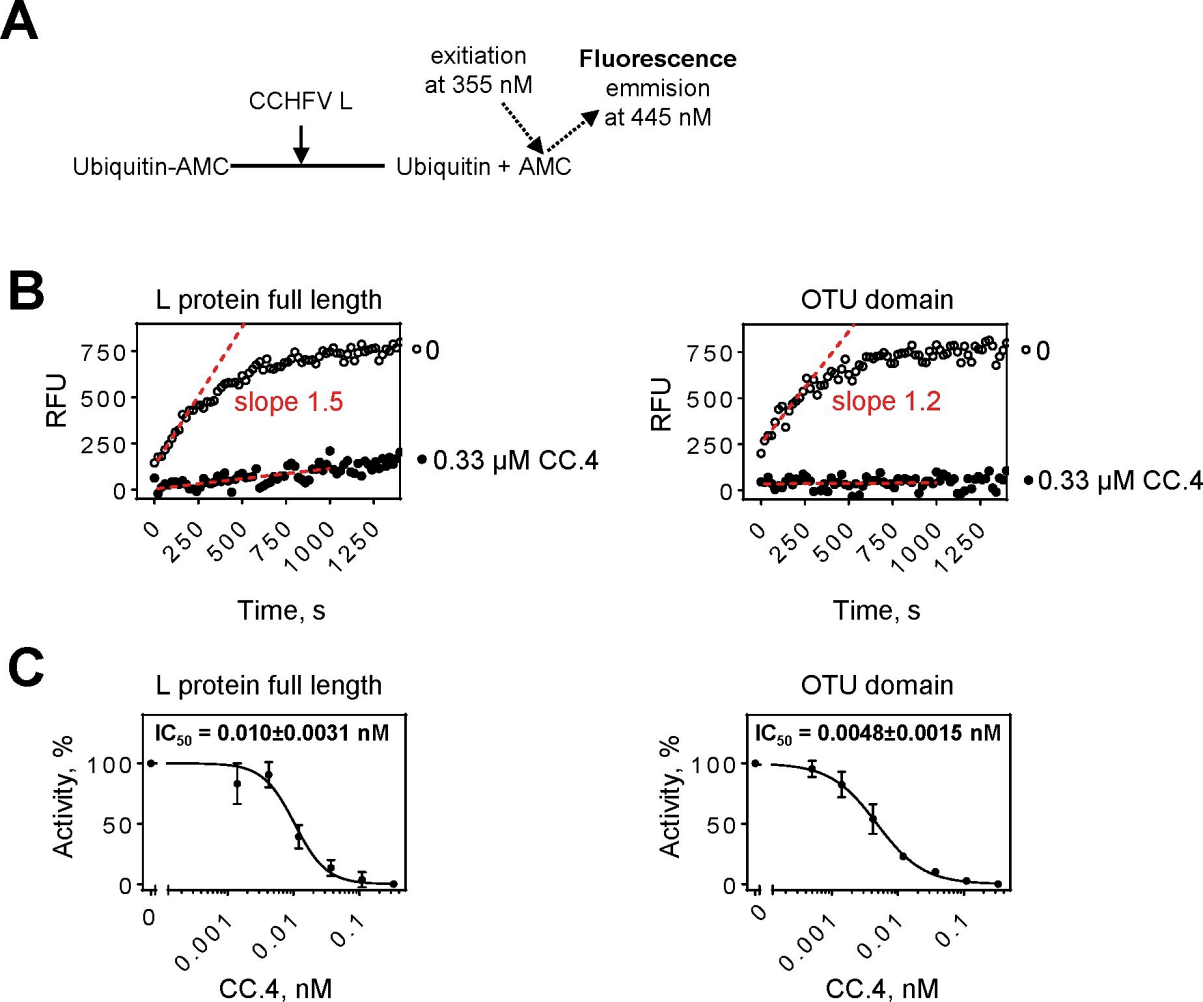
De-ubiquitinating (DUB) activity of purified CCHFV full-length L and the isolated OTU domain. (**A**) Schematic representation of the assay. A C-terminus-derived ubiquitin peptide conjugated with 4-amino-4-methylcoumarin (AMC) is used as a fluorogenic substrate for CCHFV L DUB-activity. Once AMC is released from the conjugated substrate it can emit fluorescence at 445 nn upon excitation at 355 nm. (**B**) Time dependent formation of the fluorescence signal in reactions containing ~5 nM CCHFV L protein or the isolated OTU domain and 500 nM ubiquitin-AMC substrate. Slopes (red dotted lines) of linear portions of signal formation were used to determine the velocity of substrate cleavage. DUB activity of both full length protein and OTU domain is inhibited by ubiquitin variant CC.4. (**C**) Ubiquitin variant CC.4 inhibits DUB activity of both full length protein and OTU domain to a similar extent as illustrated by the 50% inhibitory concentrations (IC_50_).

We then employed a fluorescence-based assay to translate these findings into quantitative terms (Fig 8). The assay involves the fluorogenic substrate ubiquitin-AMC that is released in the presence of DUB activity as schematically shown in Fig 8A [38]. Here we show that the CCHFV L full-length protein and the isolated OTU domain cleave ubiquitin-AMC in a time dependent manner with similar velocities illustrated by the slopes of the initial linear portion of product formation (Fig 8B). Addition of OTU-specific inhibitor CC.4 blocked DUB activity of both enzymes. IC_50_ values for the inhibition of corresponding DUB activities are 0.010±0.0031 nM and 0.0048±0.0015 nM, respectively (Fig 8C). The minor 2-fold difference suggests that the isolated OTU domain is functionally equivalent to the OTU domain of the full-length L-protein.

**Fig 9:**
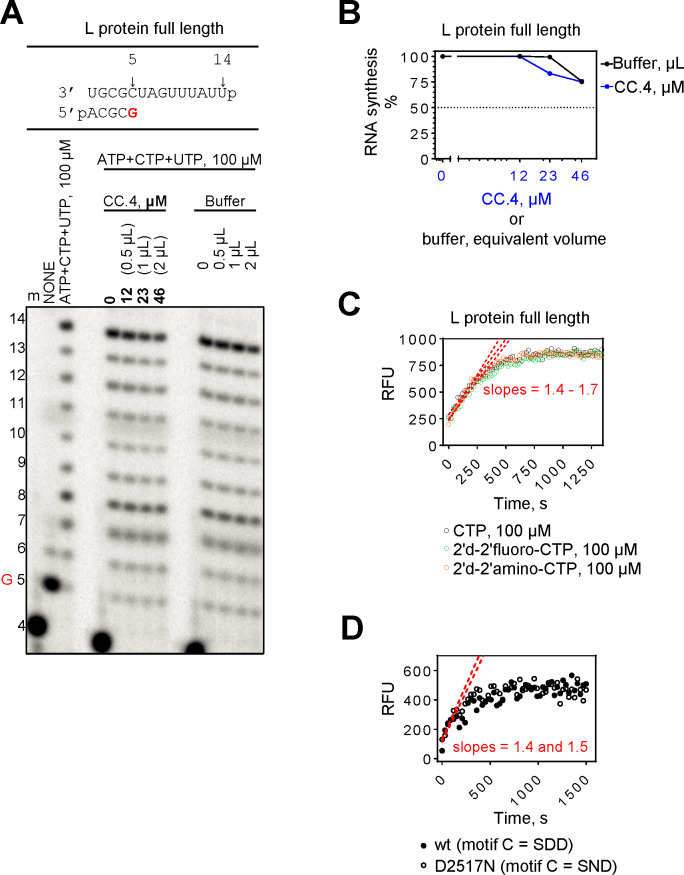
Polymerase and de-ubiquitinating (DUB) activities of purified CCHFV full-length L and the isolated OTU domain in the presence of respective inhibitors. (**A**) Ubiquitin variant CC.4 does not inhibit polymerase activity of CCHFV L protein. (**B**) Graphic representation of data shown in (**A**). (**C**) The presence of NTP substrates and nucleotide analogue inhibitors do not affect DUB activity. (D) DUB activity of CCHFV L protein wt and D2517N mutant.

To study directly a putative interdependency of RdRp and DUB activities, we compared formation of the 14-mer full-length RNA product in absence and presence of CC.4 (Fig 9). The data show that that ~900-fold excess of CC.4 over the enzyme concentration generated only a 25% decrease in the levels of RNA synthesis, which is comparable to the effect generated by the equivalent volume of the buffer only (Fig 9A and 9B). To study the putative effect of RNA synthesis or RNA synthesis blockage on the OTU domain, we monitored DUB activity in the presence of all four NTPs in the absence and in the presence of nucleotide analogues 2’-deoxy-2’-fluoro-CTP and 2’-deoxy-2’-amino-CTP. Inhibition of DUB activity is not seen under these conditions (Fig 9C). Moreover, the RdRp active site mutant D2517N retained DUB activity (Fig 9D). Together the data suggest that RdRp and DUB activities are functionally independent.

## Discussion

Effective treatments for CCHFV infection are currently not available and the requirement for high biosafety measures hamper drug discovery and development efforts with infectious virus. The identification of small molecule compounds that inhibit viral RNA synthesis relies on the availability of recombinant L protein, which is a multifunctional protein with a high molecular weight of ~450 kDa. Here we report the expression of full-length CCHFV L protein that possess active RdRp and DUB domains.

The initiation of RNA synthesis associated with segmented negative sense RNA viruses is a sequence-dependent process that commonly involves conserved regions at the 5’ and 3’ termini in the untranslated regions of the genome [17,18,39,40]. As RNA synthesis proceeds the RdRp enzyme is expected to form an elongation complex capable of transcribing the RNA template largely independent of the sequence [41]. We devised model primer/template substrates that mimic the elongation state. Gel-based biochemical assays demonstrate that the CCHFV full-length L protein is able to extend a short 4-mer primer in the presence of either Mg^2+^ or Mn^2+^. The divalent metal ions likely catalyze the nucleotidyl transfer following a two-metal ion mechanism as described for other polymerases [42,43]. The presence of all four NTPs yielded a full-length RNA product corresponding to the length of the 14-mer template. Limited RNA synthesis in the presence of only one, two, and three NTPs yielded the expected shorter products. Hence, the use of a heteropolymeric primer/template substrate allowed us to study the efficiency and fidelity of single nucleotide incorporation events. In contrast, *de novo* initiation of RNA synthesis in the absence of a primer or the use of shorter RNA primers can lead to multiple reaction products [44,45], which makes it difficult to translate the data into quantitative terms.

As demonstrated for other polymerases [5,6,13], the CCHFV L-protein can accommodate favipiravir-TP and ribavirin-TP. Both inhibitors act as ATP- or GTP-analogues. Once incorporated, these compounds compromise further incorporation events and cause partial chain-termination. However, the efficiency of incorporation of the natural counterparts is evidently favored. Incorporation of ATP (GTP) over ribavirin-TP is approximately 300- (700-)fold more efficient, which likely provides a significant competitive advantage under biologically relevant conditions. The high selectivity for natural nucleotide pools prevents frequent inclusions of the inhibitor into the growing RNA chain. This bottleneck provides a plausible explanation for the lack of any clinical benefit with ribavrin. Selectivity values for ATP(GTP) over favipiravir-TP are slightly more favorable (~90 (330)). Favipiravir was also shown to exceed the antiviral effects of ribavirin in a mouse model and in cell-based assays using a CCHFV reporter virus [11,12]. 2’-deoxy-2’-fluorocytidine is another broad spectrum antiviral that was previously identified and assessed in the same assay [15]. This nucleotide analogue showed the lowest EC_50_ value of all three compounds. 2’-deoxy-2’-fluorocytidine-TP also shows higher rates of incorporation in our biochemical assay. Incorporation of CTP over the inhibitor is much more favorable as compared to ribavirin and favipiravir. We measured a selectivity value of ~23. Of note, we identified 2’-deoxy-2’-aminocytidine-TP as a new inhibitor of the CCHFV L-protein and this compound showed the most favorable selectivity value (~15). It is currently unknown whether the non-phosphorylated mother compound or any derivative may also affect CCHFV replication. Structural data with the RdRp of human norovirus, which is a positive-sense RNA virus (*Caliciviridae* family), showed that the compound binds to the active site and causes reorientations of several amino acids [46].

The OTU domain of the CCHFV L-protein is another attractive target for the development of intervention strategies [27]. Here we demonstrate that the full-length enzyme and the isolated OTU domain show very similar levels of DUB activities. Both enzymes cleave Lys48- and Lys63-linked polyubiqutin chains. The ubiquitin variant CC.4 also inhibits both reactions. These results enabled us to study the relationship or possible interdependency of RdRp and OTU domains. First of all, the OTU protease activity is not required to obtain active RdRp enzyme. SDS-PAGE and MS analysis revealed a single band that was identified as the full-length CCHFV L protein with > 95% peptide coverage. Autocatalytic processing is not observed and the OTU domain remains attached to the RdRp domain. RNA synthesis mediated by the L-protein was previously also demonstrated in cell-based minigenome systems [10]. Western analysis likewise revealed that the L-protein is not further processed by the OTU domain. A mutation in the OTU catalytic site (C40A) did not affect minigenome replication suggesting that the OTU protease activity of the L-protein is not required for RNA synthesis. Conversely, Scholte and colleagues reported more recently that the same mutation abolished viral propagation [26]. The presence of the OTU inhibitor CC.4 also caused inhibition of CCHFV replication in addition to enhancing host antiviral responses [28]. The authors considered different scenarios that help to reconcile this data. The interaction between the OTU domain and CC.4 may affect formation of higher order complexes containing RNA, L-protein and the nucleoprotein (NP). Alternatively, the bound inhibitor may render the polymerase inactive. Our biochemical data show that the presence of CC.4 does not affect elongation of RNA synthesis. Moreover, inhibition of RdRp activity in the presence of nucleotide analogues does not affect DUB activity. These results demonstrate that RdRp and OTU activities are functionally independent. However, it is conceivable that the ubiquitin variant affects the intracellular recruitment of other host or viral factors to the replication complex. We can also not exclude that DUB activity and the specific initiation of RNA synthesis are somehow linked. Structural data with FluB RdRp revealed domain rearrangements that may also play a role for the CCHFV RdRp during initiation [19,47].

## Conclusion

The expression of recombinant full-length CCHFV L-protein provides an important tool for novel drug discovery and development efforts. Screening of large libraries of small molecules is now feasible, given that biochemical RdRp assays do not require high biosafety measures. Non-nucleoside inhibitors of CCHFV RdRp are currently unknown and high-throughput screening (HTS) campaigns have the potential to identify such compounds [48,49]. For this purpose, the gel-based assays utilized in this study need be transferred ideally into plate-based assays with a non-radioactive readout [50]. The few known nucleotide-like inhibitors against CCHFV show a broad spectrum of activities against several RNA viruses. The availability of the full-length L-protein enables the discovery of more selective compounds with a greater competitive advantage over natural nucleotide pools. Although DUB activities in the context of the full-length L-protein and the isolated domain are very similar, it is conceivable that the structural environment of the L-protein may also facilitate the discovery of small molecule inhibitors with high selectivity over human DUB orthologs.

## Supporting information

S1 Fig Summary of the CCHFV L protein mass-spectroscopy data. Portions of the CCHFV L protein sequence that have been detected by mass-spectroscopy are highlighted in yellow. The raw data file can be accessed by clicking here.
(DOCX)

S2 Fig Snapshots of the CCHFV L protein mass-spectroscopy data for wt and D2517N mutant. The raw data files can be accessed by clicking here.
(TIF)
