# Supplementary material for: Independent inhibition of the polymerase and deubiquitinase activities of the Crimean-Congo Hemorrhagic Fever Virus full-length L-protein
Source: PLoS Negl Trop Dis. 2020 Jun 4;14(6):e0008283. doi: 10.1371/journal.pntd.0008283 (PMC7271988; doi:10.1371/journal.pntd.0008283)
Supplement: S1 Fig — Portions of the CCHFV L protein sequence that have been detected by mass-spectroscopy are highlighted in yellow. The raw data file can be accessed by clicking here. (DOCX) [file pntd.0008283.s001.docx]

**>AIE16126.1 CCHFV L protein**

MNFLRNLDWTQVIAGQYVSNPRFNISDYFEIVRQPGDGNCFYHSIAELTMPNKTDHSYHNIKRLTESAAR

KYYQEEPEAGLVGLSLEDYLKRMLSDNEWGSTLEASMLAKEMGITIIIWTVAASDEVEAGIKFGDGDVFT

AVNLLHSGQTHFDALRILPQFEADTRETLSLMDRVIAVDQLTSSSSDELQDYEDLALALTSAEESYRRSS

LDEVTLSKKQAEILRQKASQLSKLVNKSQNIPTRVGRVLDCMFNCKLCVEISADTLILRPESKEKIGEIM

SLRQLGHKLLTRDKQIKQEFSRMKLYVTKDLLDHLDVGGLLRAAFPGTGIERHMQLLHSEMILDICTVSL

GVMLSTFLYGSNNKNKKKFITNCLLSTALSGKKVYKVLGNLGNELLYKAPRKALATVCSALFGKQINKLQ

NCFRTISPVSLLALRNLDFDCLSVQDYNGMIENMSKLDNTDVEFNHREIADLNQLTSRLITLRKEKDTDL

LKQWFPESDLTRRSTRNAANAEEFVISEFFKKKDIMKFISTSGRAMSAGKIGNVLSYAHNLYLSKSSLNM

TSEDISQLLIEIKRLYALQEDSEVEPIAIICDGIESNMKQLFAILPPDCARECEVLFDDIRNSPTHSTAW

KHALRLKGTAYEGLFANCYGWQYIPEDIKPSLTMLIQTLFPDKFEDFLDRTQLHPEFRDLTPDFSLTQKV

HFKRNQIPSVENVQISIDATLPESVEAVPVTERKMFPLPETPLSEVHSIERIMENFTRLMHGGKLSAKKK

DGDPAGQDSQQSTTEHESTSISAFKDYGERGIVEENHMRFSEEDQLETRQLLLVEVGFQTDIDGKIRTDH

KKWKDILKLLELLGIKCSFIACADCSSTPPDRWWISEDRVRVLKNSVSFLFNKLSRNSPTEVTDIVVGAI

STQKVRSYLKAGTATKTPVSTKDVLETWEKMKEHILNRPTGLTLPTSLEQAMRKGLVEGVVISKEGSESC

INMLKENLDRITDEFERTKFKHELTQNITTSEKMLLSWLSEDIKSSRCGECLSSIKKTVDETANLSEKIE

LLAYNLQLTNHCSNCHPNGLNISNTSNVCKRCPKIEVVSHCENKGFEDSNECLTDLDRLVRLTLPGKTEK

ERRVKRNVEYLIKLMMSMSGIDCIKYPTGQLITHGRVSAKHNDGNLKDRSDDDQRLAEKIDAVRKELSES

KLKDYSTYAKGVISNSLKNLSRQGKSKCSVPRSWLEKILFDLKVPTRDEEVLINIRNSLKARSEFVRNND

KLLIRSKEELKKCFDVQSFKLKKNKQPVPFQVDCILFKEVAAECMKRYIGTPYEGIVDTLVSLINVLTRF

TWFQEVVLYGKICETFLRCCTEFNRSGVKLVKIRHCDINLSVKLPSNKKENMLCCLYSSNMELLQGPFYL

NRRQAVLGSSYLYIVITLYIQVLQQYRCLEVINSVNEKTLQDVENHSMTLLEDSFKEITFALEGRFEESY

KIRTSRCRASGNFLNRSSRDHFISVVSGLNLVYGFLIKDNLLANSQQQNKQLQMLRFGMLAGLSRLVCPN

ELGKKFSTSCRRIEDNIARLYLQTSIYCSVRDVEDNVKHWKQRDLCPEVTIPCFTVYGTFVNSDRQLIFD

IYNVHIYNKEMDNFDEGCISVLEETAERHMLWELDLMNSLCSDEKRDTRTARLLLGCPNVRKAANREGKK

LLKLNSETSTDTQSIASEVSDRRSYSSSKSRIRSIFGRYNSQKKPFELRSGLEVFNDPFNDYQQAITDIC

QFSEYTPNKESILKDCLQIIRKNPSHTMGSFELIQAISEFGMSKFPPENIDKARRDPKNWVSISEVTETT

SIVASPRTHMMLKDCFKIILGTENKKIVKMLRGKLKKLGAISTNIEIGKRDCLDLLSTVDGLTDQQKENI

VNGIFEPSKLSFYHWKELVRKNIDEVLLTEDGNLIFCWLKTISSSVKGSLKKRLKFMNVHSPELMPENCL

FSSEEFNELIKLKRLLLNEQQDEQELKQDLLISSWIKCITACKDFASINDKIQKFIYHLSEELYDIRLQH

LELSKLKQEHPSVSFTKEEVLIKRLEKNFLKQHNLEIMETVNLIFFAALSAPWCLHYKALESYLVRHPEI

LDCGSKEDCKLTLLDLSVSKLLVCLYQKDDEELTNSSSLKLGFLVKYAVTLFTSNGEPFSLSLNDGGLDL

DLHKTTDEKLLHQTKIVFAKIGLSGNSYDFIWTTQMIANSNFNVCKRLTGRSTGERLPRSVRSKVIYEMV

KLVGETGMAILQQLAFAQALNYEHRFYAVLAPKAQLGGARDLLVQETGTKVMHATTEMFSRNLLKTTSDD

GLTNPHLKETILNVGLDCLANMRNLDGKPISEGSNLVNFYKVICISGDNTKWGPIHCCSFFSGMMQQVLK

NVPDWCSFYKLTFIKNLCRQVEIPAGSIKKILNVLRYRLCSKGGVEQHSEEDLRRLLTDNLDSWDGNDTV

KFLVTTYISKGLMALNSYNHMGQGIHHATSSVLTSLAAVLFEELAIFYLKRSLPQTTVHVEHAGSSDDYA

KCIVVTGILSKELYSQYDETFWKHACRLKNFTAAVQRCCQMKDSAKTLVSDCFLEFYSEFMMGYRVTPAV

IKFMFTGLINSSVTSPQSLMQACQVSSQQAMYNSVPLVTNTAFTLLRQQIFFNHVEDFIRRYGILTLGTL

SPFGRLFVPTYSGLVSSAVALEDAEVIARAAQTLQMNSVSIQSSSLTTLDSLGRCRTSSTAEDSSSVSDT

TAASHDSGSSSSSFSFELNRPLSETELQFIKALSSLKSTQACEVIQNRITGLYCNSNEGPLDRHNVIYSS

RMADSCDWLKDGKRRGNLELANRIQSVLCILIAGYYRSFGGEGTEKQVKASLNRDDNKIIEDPMIQLIPE

KLRRELERLGVSRMEVDELMPSISPDDTLAQLVAKKLISLNVSTEEYSAEVSRLKQTLTARNVLHGLAGG

IKELSLPIYTIFMKSYFFKDNVFLSLTDRWSTKHSTNYRDSCGKQLTGRIITKYTHWLDTFLGCSVSINR

HTTVKEPSLFNPNIRCVNLITFEDGLRELSVIQSHLKVFENEFTNLNLQFSDPNRQKLRIVESRPAESEL

EANRAVIVKTKLFSATEQVRLSNNPAVVMGYLLDESAISEVKPTKVDFSNLLKDRFKIMQFFPSVFTLIK

MLTDESSDSEKNGLSPDLQQVARYSNHLTLLSRMIQQAKPTVTVFYMLKGNLMNTEPTVAELVSYGIKEG

RFYRLSDTGIDASTYSVKYWKILHCISAIGCLPLSQADKSSLLMSFLNWRVNMDIRTSDCPLSSHEASIL

SEFDGQVIANILASELSSVKRDSEREGLTDLLDYLNSPTELLKKKPYLGTTCKFNTWGDSNRSGKFTYSS

RSGESIGIFIAGKLHIHLSSESVALLCETERQVLSWMSKRRTEVITKEQHQLFLSLLPQSHECLQKHKDG

SALSVIPDGSNPRLLKFVPLKKGLAVVKIKKQILTVKKQVVFDAESEPRLQWGHGCLSIVYDETDTQTTY

HENLLKVKHLVDCSTDRKKLLPQSVFSDSKVVLSRIKFKTELLLNSLTLLHCFLKHAPSDAIMEVESKSS

LLHKYLKSGGVRQRNTEVLFREKLNKVVIKDNLEQGVEEEIEFCNNLTKTVSENPLPLSCWSEVQNYIED

IGFNNVLVNIDRNTVKSELLWKFTLDTNVSTTSTIKDVRTLVSYVSTETIPKFLLAFLLYEEVLMNLINQ

CKAVKELINSTGLSDFELESLLTLCAFYFQNECSKRDGPRCSFAALLSLVHEDWQRIGKNILVRANNELG

DVSLKVNIVLVPLKDMSKPKPERVVMARRSLNHALSLMFLDEMSLPELKSLSVNCKMGNFEGQECFEFTI

LKDNSARLDYNKLIDHCVDMEKKREAVRAVEDLILMLTGKAIKPSAVTQFVHEDEQCQEQISLDDLMAND

TVTDLPDREAEALRTGNLGFNWDSD
