# Supplementary figures and images for: Independent inhibition of the polymerase and deubiquitinase activities of the Crimean-Congo Hemorrhagic Fever Virus full-length L-protein
Source: PLoS Negl Trop Dis. 2020 Jun 4;14(6):e0008283. doi: 10.1371/journal.pntd.0008283 (PMC7271988; doi:10.1371/journal.pntd.0008283)

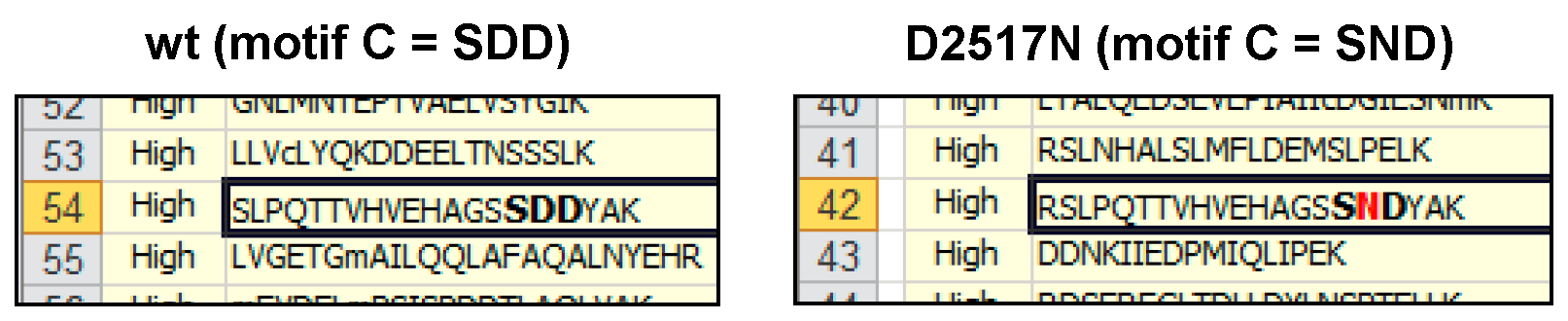

Supplement: S2 Fig — The raw data files can be accessed by clicking here. (TIF) [file pntd.0008283.s002.tif]
